# Practice patterns and determinants of wait time for autism spectrum disorder diagnosis in Canada

**DOI:** 10.1186/s13229-018-0201-0

**Published:** 2018-03-06

**Authors:** Melanie Penner, Evdokia Anagnostou, Wendy J. Ungar

**Affiliations:** 1 Autism Research Centre, Bloorview Research Institute, Holland Bloorview Kids Rehabilitation Hospital, Toronto, Canada; 2 Department of Paediatrics, University of Toronto, Toronto, Canada; 3 Technology Assessment at Sick Kids (TASK), Child Health Evaluative Sciences, The Hospital for Sick Children Research Institute, Toronto, Canada; 4 Institute of Health Policy, Management and Evaluation, University of Toronto, Toronto, Canada

**Keywords:** Autism spectrum disorder, Diagnosis, Early detection, Pediatrics, Health services research

## Abstract

**Background:**

Inefficient diagnostic practices for autism spectrum disorder (ASD) may contribute to longer wait times, delaying access to intervention. The objectives were to describe the diagnostic practices of Canadian pediatricians and to identify determinants of longer wait time for ASD diagnosis.

**Methods:**

An online survey was conducted through the Canadian Paediatric Society’s developmental pediatrics, community pediatrics, and mental health sections. Participants were asked for demographic information, whether they diagnosed ASD, and elements of their diagnostic assessment. A multiple linear regression of total wait time (time from referral to communication of the diagnosis to the family) as a function of practice characteristics was conducted.

**Results:**

A total of 90 participants completed the survey, of whom 57 diagnosed ASD in their practices (63.3%). Respondents reported varied use of multi-disciplinary teams, with 53% reporting participation in a team. No two identically composed teams were reported. Respondents also had varied use of diagnostic tools, with 21% reporting no use of tools. The median reported total wait for ASD diagnosis time was 7 months (interquartile range 4–12 months). Longer time spent on assessment was the only variable that remained significantly associated with longer wait time in multiple regression (*p* = 0.002). Use of diagnostic tools did not significantly affect wait time.

**Conclusion:**

Canadian ASD diagnostic practices vary widely and wait times for these assessments are substantial—7 months from referral to receipt of diagnosis. Time spent on the assessment is a significant determinant of wait time, highlighting the need for efficient assessment practices.

**Electronic supplementary material:**

The online version of this article (10.1186/s13229-018-0201-0) contains supplementary material, which is available to authorized users.

## Background

Autism spectrum disorder (ASD), a neurodevelopmental disorder defined by impairment in social communication and the presence of restricted repetitive behaviors [1], has steadily increased in reported prevalence over the past decade [2]. Pediatricians are frequently involved in ASD diagnosis in pre-school age children in Canada [3]. Numerous guidelines have been published for diagnostic assessment for ASD, with varied recommendations for personnel and tools in the assessment [4–9]. A concern about models for diagnostic assessment is how they may extend the waiting period to receive intervention. Waiting for an ASD diagnostic assessment occurs during a critical period of brain development [10] and an extended wait time may delay receipt of intervention and reduce effectiveness [11]. To date, little work has been done looking specifically at wait times for ASD diagnosis, and how diagnostic practices influence wait times.

Age at diagnosis has often been used as a proxy to understand wait times; however, other factors beyond diagnostic demand and supply influence this metric. Milder ASD subtypes have been associated with a later age at diagnosis [12]. These children may not show significant impairment associated with ASD until their skills are exceeded by social demands, which will occur later for more mildly affected children. Additional studies have shown that severe language impairment/regression, unusual mannerisms, and toe walking were features of the clinical presentation associated with younger age at diagnosis [13, 14]. Co-occurring or alternative diagnoses such as attention deficit hyperactivity disorder may delay diagnostic evaluation for ASD [15]. Factors external to the child also decrease the age at diagnosis, including older maternal age [15] and having relatives with ASD [14], both of which may be indicative of caregivers who are more aware of the possibility of ASD. Lower socioeconomic status, being a visible minority, and living in a rural setting are all associated with an older age at diagnosis [13, 14, 16]. The number of potential confounders makes it difficult to isolate the impact of wait times for assessment on the diagnosis age.

Few studies have reported current ASD diagnostic practices and their association with wait times. One USA study evaluated factors related to wait times for diagnosis, which was 13 months on average in their sample [17]. Reported associations in this study were mostly between wait times and patient demographic factors, but there was no association between the use of a standardized diagnostic tool and mean age of first ASD diagnosis. One chart review of 70 children’s cases from Scotland found that receiving more information prior to the assessment, such as contextual information and results of other assessments, reduced the number of assessment visits needed and decreased the total wait for diagnosis [18]. This sample was taken from only eight children’s services, and therefore provides little insight on between-provider variability in practice and its impact on wait times.

Further information on diagnostic assessment and wait times from diverse clinical practices is needed to inform ASD service planning in constrained health care systems. The study objectives were to (1) document the diversity of practices of Canadian pediatricians with regard to their diagnostic assessment for ASD; and (2) to identify the elements of clinical diagnostic practice that are associated within a longer wait times for ASD diagnosis.

## Methods

### Study design

This was a cross-sectional survey of pediatricians across Canada to investigate ASD diagnostic practices. The study was approved by the Research Ethics Board of The Hospital for Sick Children and participants were informed that survey completion indicated their consent.

### Target population

The Canadian Paediatric Society (CPS) is the national association of Canadian pediatricians, representing over 3000 pediatricians and pediatric trainees [19]. In Canada, most primary care is provided by family physicians, who consult pediatricians if the child’s care needs exceed their scope of practice. Developmental pediatricians are subspecialists, some of whom only accept referrals from pediatricians, necessitating multiple referrals before reaching this level of expertise. There are no uniform ASD diagnostic requirements across Canada, making it an ideal setting to explore varying diagnostic practices and their impact on wait times. Three sections were chosen for survey distribution based on their likelihood of participating in ASD diagnosis: developmental pediatrics, community pediatrics, and mental health. Current members of these sections who were practicing pediatrics in Canada and who were able to complete the survey in English were eligible to participate.

### Survey administration

The survey instrument was designed based on a review of the literature and clinical experiences of the researchers (Additional file 1). The survey collected information on provider demographics, (age, sex, province, catchment area, years in pediatric practice, type of health professional, training in child development). For those that diagnosed ASD in their practice, the survey asked about the participants’ current wait time in months for the first visit of the diagnostic assessment and the current wait time in weeks from the first assessment visit to communication of the ASD diagnosis to the family. The survey was piloted in November 2014 with two Ontario developmental pediatricians and two general pediatricians. Minor changes were made to improve clarity. The main survey was administered in March 2015. Using an electronic mail list serve, the CPS sent all potential participants an email containing an online link to complete an electronic version of the survey created using REDCap (Research Electronic Data Capture) [20]. Participants had 3 weeks to complete the survey, during which they were sent two reminder emails.

### Statistical analysis

**Fig. 1:**
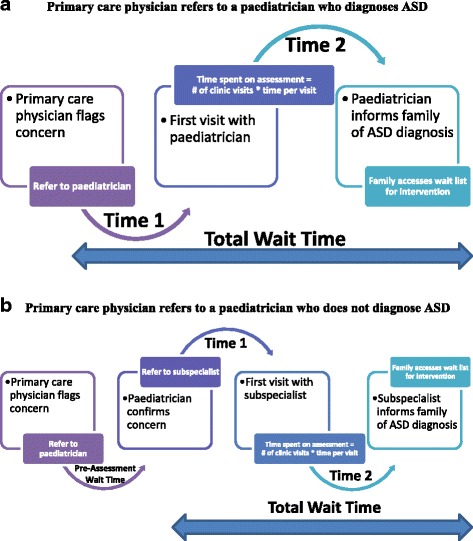
These figures show **a** the referrals and wait times for a child with suspected ASD from a primary care physician to a pediatrician who diagnoses ASD; and **b** the referrals and wait times for a child suspected ASD initially referred from a primary care physician to a pediatrician who does not diagnose ASD and subsequently refers to a subspecialist. Note that the total wait time does not include the pre-assessment wait time (time for consultation with pediatrician who does not diagnose ASD). # = number; * = multiplied by

All data were exported from the online database to R for statistical analysis (Vienna, Austria, 2014). Descriptive statistics were calculated for all question responses. Demographic characteristics for participants who diagnose ASD were compared to those who do not diagnose ASD using non-parametric statistics. The median amount billed per clinic visit was calculated. Time-based billing codes were excluded from the analysis as the total amount billed per visit could not be calculated without knowledge of the amount of time spent on each visit. Wait times for ASD diagnosis are defined in Fig. 1. The wait time between receiving the referral and the first scheduled visit of the diagnostic assessment (time 1) was reported in months and converted to days in the analysis. The wait time from the first visit of the diagnostic assessment to the communication of the diagnosis to the family (time 2) was reported in weeks and converted to days for the analysis. The total wait time (in days) was calculated for each participant by adding time 1 and time 2. Times are reported in months for ease of interpretation.

A multiple linear regression of total wait time as a function of diagnostic assessment characteristics was conducted. A subspecialized assessor (a developmental pediatrician) and practicing in a multi-disciplinary team (MDT) were each hypothesized to be independently positively correlated with a longer wait time due to constraints on availability. Province of practice was included as a covariate, as provincial policies may dictate necessary elements of the assessment [4]. The clinician’s catchment area was included because a larger service population may increase wait times [14]. Accepting referrals from family physicians was hypothesized to be associated with longer wait times due to a higher volume of referrals for assessment. More junior clinicians have been reported to work fewer hours [21, 22], which may increase their wait times.

Time spent on assessment was calculated for each participant by multiplying the number of diagnostic visits by the average reported length of each visit selected from a list of options for visit length (Additional file 1); the mid-range value was taken as the average length of each visit. Longer time spent on assessment was hypothesized to be positively correlated with wait time due to fewer clinic slots available for new assessments.

A series of bivariate analyses to test the association between hypothesized covariates and total wait time were initially performed to determine which explanatory variables to include in the regression model. The significance level for inclusion in the model was set at 0.2. Because none of the variables were normally distributed, non-parametric tests including Spearman correlation for continuous variables and Kruskal-Wallis and Wilcoxon tests for categorical variables were used to determine the significance of the observed associations. Non-parametric tests were used to determine the relationship between all potential covariates. Two variables were considered to be collinear when they were significantly associated (*p* < 0.05); these variables were not tested together in the model. The dependent variable and each of the variables that were significant in the bivariate analyses were entered one by one to build a forward multivariate linear regression model. If a variable was significantly correlated with the dependent variable (*p* < 0.05), it remained in the model. As the dependent variable total wait time was skewed, it was transformed to a natural logarithm (ln), with normal distributions of the residuals confirmed for the ln-transformed analyses. Goodness of fit was tested with *R*^2^. Back transformation of the ln-transformed dependent variable was performed by calculating the Duan smearing estimate [23]. The predicted values of wait time were multiplied by the smearing estimate to determine the mean adjusted wait times (with 95% confidence intervals) based on the included covariate(s).

An additional analysis was conducted to assess the association between wait time and diagnostic tool use. The total wait time was compared between respondents who did and did not report use of various diagnostic tools using Wilcoxon tests.

## Results

Of 639 individuals solicited, 91 responses were received (response rate of 14%). One participant who was a speech language pathologist (not a pediatrician) was removed from the sample, leaving 90 respondents. Eighty-five respondents completed all mandatory questions (5 incomplete). The demographic information for the total sample is displayed in Table 1. A majority of participants (66%) were female. The sample included representation from all Canadian provinces with the exception of PEI, and from two territories (no responses from the Northwest Territories), with a proportionally larger representation from Ontario.

**Table 1:** Demographics and practice patterns

Characteristic	Total sample (*n* = 90)		Diagnose ASD (*n* = 57)		Do not diagnose ASD (*n* = 33)		*p*
	*n*	%	*n*	%	*n*	%	
Age (years), median; range; IQR	54; 29–77; 42–63		52; 29–77; 41–60		57; 31–76; 45–64		0.17^a^
Years in practice, median; range; IQR	21; 0.5–46; 8–30.5		18; 0.5–46; 7–28		26; 1–44;13–35		0.08^b^
Sex							
Male	30	33.3%	19	33.3%	11	33.3%	
Female	60	66.7%	38	66.7%	22	66.7%	1
Province of practice							
Ontario	39	43.3%	27	47.4%	12	36.3%	
Alberta	15	16.7%	11	19.3%	4	12.1%	
Quebec	12	13.3%	4	7%	8	24.2%	
British Columbia	6	6.7%	3	5.3%	3	9.1%	
New Brunswick	4	4.4%	4	7%	–	–	
Newfoundland and Labrador	4	4.4%	2	3.5%	2	6.1%	
Nova Scotia	4	4.4%	1	1.8%	3	9.1%	
Manitoba	2	2.2%	1	1.8%	1	3%	
Saskatchewan	2	2.2%	2	3.5%	–	–	
Nunavut	1	1.1%	1	1.8%	–	–	
Yukon	1	1.1%	1	1.8%	–	–	0.14^c^
Catchment							
Within regional health authority	28	31.1%	20	35.1%	8	24.2%	
Within city only	23	25.6%	15	26.3%	8	24.2%	
Within province/territory	20	22.2%	15	26.3%	5	15.1%	
No defined catchment	17	18.9%	7	12.3%	10	30.3%	
Missing	2	2.2%	–	–	2	6.1%	0.07^c^
Type of professional							
General pediatrician	64	70%	33	57.9%	31	93.9%	
Developmental pediatrician	23	25.6%	22	38.6%	1	3%	
Neonatologist	2	2.2%	1	1.8%	1	3%	
Pediatrician + allergist	1	1.1%	1	1.8%	–	–	< 0.01^c^
Extra training in child development							
None	57	63.3%	28	49.1%	29	87.9%	
Fellowship in developmental pediatrics	22	24.4%	22	38.6%	–	–	
General pediatrics training with additional child development	5	5.6%	3	5.3%	2	6.1%	
Continuing medical education	2	2.2%	1	1.8%	1	3%	
Fellowship in pediatric neurology	2	2.2%	2	3.5%	–	–	
Participation in a MDT	1	1.1%	1	1.8%	–	–	
Missing	1	1.1%	–	–	1	3%	< 0.01^c^

*IQR* interquartile range, *MDT* multi-disciplinary team; percentages may not sum to 100% due to rounding

^a^Wilcoxon rank sum test *W* = 803

^b^Wilcoxon rank sum test *W* = 758

^c^Using Fisher’s exact test

### Practice characteristics

Practice characteristics were summarized separately for those who did not diagnose ASD (*n* = 33) and those who did (*n* = 57) and are compared in Table 1. There were no statistically significant differences between these two groups in their ages, years in practice, sex, province of practice, or catchment areas. Significant differences were seen between the two groups in the types of professionals, with all but one developmental pediatrician indicating they diagnose ASD, and in additional training in child development, where all participants who had undertaken a developmental pediatrics fellowship were in the diagnosing group.

Participants who did not diagnose ASD were pediatricians who would either confirm or refute a developmental concern raised by a primary care physician (Fig. 1). The median wait time for consultation with this group (pre-assessment wait time, Fig. 1b) was 70 days (range 14 to 560), with 60.1% of participants reporting a visit length of 46–75 min. The median amount billed for a developmental consultation was $171.82 (full list of reported billing codes for this group is presented in Appendix 1).

General information about ASD diagnosticians is reported in Table 2. Fifteen (26.3%) respondents provided a definitive assessment in more than half of their cases, although responses were missing for 44% of participants for this optional question. The majority of respondents (87.7%) reported that they referred to regional developmental intervention services for some or all of their assessments thereby obtaining input from other disciplines. Commonly ordered tests included hearing (80.7% of respondents ordered this in the majority of assessments), chromosomal microarray (68.4%), and Fragile X (64.9%).

**Table 2:** Practice patterns for participants who diagnose ASD (*n* = 57)

Practice characteristic	*n*	%
Accepts referrals from family doctor		
Yes	44	77.2%
No	12	21.1%
Missing	1	1.8%
Number of visits to make a diagnosis of ASD		
1 visit	8	14.0%
2 visits	33	57.9%
3 visits	11	19.3%
4 visits	4	7.0%
5 visits	1	1.8%
Reported typical visit length		
< 30 min	3	5.3%
31–60 min	20	35.1%
61–90 min	19	33.3%
91–120 min	8	14.0%
121–180 min	5	8.8%
> 180 min	2	3.5%
For what percentage of cases of ASD do you provide a definitive assessment?		
0–25%	6	10.5%
26–50%	11	19.3%
51–75%	4	7.0%
76–100%	11	19.3%
Missing	25	43.9%
Practice in MDT		
Yes	30	52.6%
No	27	47.4%
Percentage of cases assessed with MDT		
1–25%	4	7.0%
26–50%	3	5.3%
51–75%	5	8.8%
76–100%	18	31.6%
(Did not practice in team) 0%	27	47.4%
Percentage of assessments with consultation with regional speech-language pathology		
0%	1	1.8%
1–25%	17	29.8%
26–50%	12	21.1%
51–75%	9	15.8%
76–100%	17	29.8%
Missing	1	1.8%
Percentage of assessments with consultation with regional developmental early intervention staff		
0%	7	12.3%
1–25%	20	35.1%
26–50%	13	22.8%
51–75%	6	10.5%
76–100%	10	17.5%
Missing	1	1.8%
Percentage of assessments with consultation with regional occupational therapist		
0%	5	8.8%
1–25%	27	47.4%
26–50%	11	19.3%
51–75%	6	10.5%
76–100%	7	12.3%
Missing	1	1.8%
Tests ordered for the majority of assessments		
Hearing	46	80.7%
Chromosomal microarray	39	68.4%
Fragile X	37	64.9%
Vision	22	38.6%
Metabolic screening	9	15.8%
MRI brain	4	7.0%
MECP2	3	5.3%
EEG	0	–
Other^a^	2	3.5%
None	1	1.8%

^a^Text response: “Depends on presentation”; *ASD* autism spectrum disorders, *EEG* electroencephalogram, *MDT* multidisciplinary team, *MECP2* methyl cytosine phosphate guanine binding protein 2 (genetic testing for Rett syndrome), *MRI* magnetic resonance imaging; percentages may not sum to 100% due to rounding; respondents could select more than one test and these percentages will not sum to 100% as a result

A wide range of billing codes and amounts for ASD assessment was observed (Appendix 2). The first visit was associated with a median billing of $229.15 (range $47 to $411.87). The second visit had a median billing of $187 (range $76.71 to $300.70), and the third visit also had a median billing of $187 (range $92.40 to $300.70).

Participants indicated using a variety of tools in the assessment for ASD, including the Autism Diagnostic Observation Schedule (ADOS), the Autism Diagnostic Interview–Revised (ADI-R), and the Childhood Autism Rating Scale (CARS). Forty percent of participants (23 participants) used more than one tool as part of their assessment, though the most commonly reported tool employed was the ADOS alone (11 participants) followed by the ADOS and ADI-R combined (6 participants). Twelve participants (21.1%) reported using no tools in the assessment. The number of participants using each tool, along with the time spent on administration and scoring, is included in Appendix 3.

**Fig. 2:**
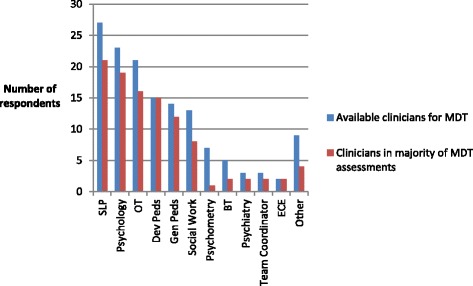
This figure shows the number of respondents indicating participation of each type of clinician available to the MDT for involvement in diagnostic assessments (blue bars) and those that participate in the majority of assessments (red bars). SLP = speech language pathology; OT = occupational therapy; Dev Peds = developmental pediatrics; Gen Peds = general pediatrics; BT = behavior therapy; ECE = early childhood educator; other is comprised of clinicians reported only once: ASD service provider, audiology, clinical genetics, dietician, family liaison, gastroenterology, neurology, neuropsychology, and nursing

Although 52.6% of diagnosticians reported practicing as part of a MDT, no two MDTs across the country had the same composition, even when limiting to team members involved in the majority of assessments. While 57% of respondents indicated that psychologists participated in the majority of diagnostic assessments, 5 of the 30 respondents belonging to MDTs did not have access to a psychologist. Speech language pathologists and occupational therapists were also frequently identified team members. The frequencies of clinicians available to MDTs, as well as those included in the majority of diagnostic assessments, are shown in Fig. 2.

### Wait times

Wait times for a first visit (time 1) varied from 1 to 24 months (interquartile range 3–9 months). Wait times for a diagnosis (time 2) also had a wide range, with half of the respondents reporting an interval of between 0.5 and 1.5 months, with some respondents indicating a much longer wait. The total wait time varied from 2 to 24 months, with a median of 7 months (Table 3).

**Table 3:** Wait times for ASD diagnostic assessment (*n* = 57)

	*n*	%	Median	Range	Interquartile range
Wait time for first visit (time 1)			6	1–24	3–9
1 month	3	5.3%			
2 months	7	12.3%			
3 months	10	17.5%			
4 months	4	7.0%			
5 months	1	1.8%			
6 months	10	17.5%			
7 months	2	3.5%			
8–9 months	5	8.8%			
10–12 months	7	12.3%			
13–18 months	5	8.8%			
19–24 months	3	5.3%			
Wait time from first visit to diagnosis (time 2)			1	0–6.5	0.5–2
0 months	7	12.3%			
0.25 months	3	5.3%			
0.5 months	5	8.8%			
0.75 months	5	8.8%			
1 month	18	31.6%			
1.5 months	2	3.5%			
2 months	7	12.3%			
3 months	4	7.0%			
4.5 months	2	3.5%			
≥ 6 months	4	7.0%			
Total wait from referral to diagnosis			7	2–26	4–12
< 2 months	3	5.3%			
2–3 months	6	10.5%			
4 months	6	10.5%			
5–6 months	7	12.3%			
7–8 months	9	15.8%			
9–10 months	8	14.0%			
11–12 months	2	3.5%			
13–14 months	6	10.5%			
15–18 months	3	5.3%			
19–22 months	4	7.0%			
23–26 months	3	5.3%			

Percentages may not sum to 100 due to rounding

**Fig. 3:**
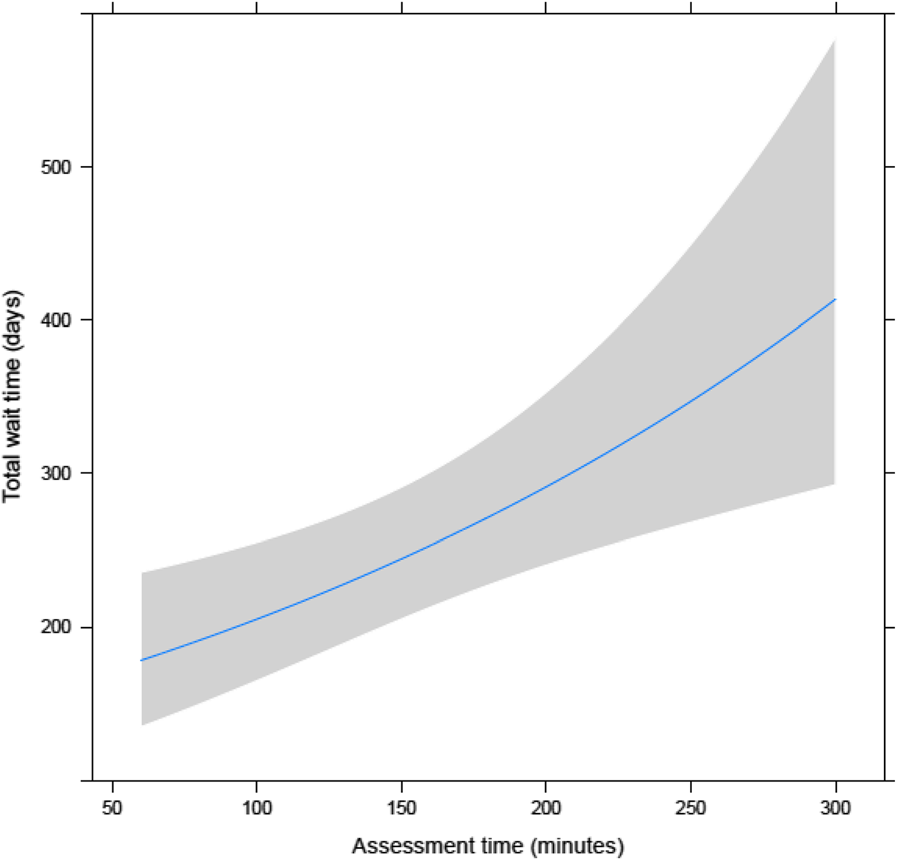
This figure shows the predicted total wait time from referral to completion of the ASD diagnostic assessment based on assessment time. Assessment time in minutes is plotted on the horizontal axis and wait time in days on the vertical axis. The blue line represents the mean adjusted value, with the shaded zone representing the 95% confidence interval

The results of bivariate analyses exploring potentially significant demographic and practice factors explaining total wait time are presented in Table 4. Time spent on assessment was significantly positively correlated with total wait time (*r* = 0.31, *p* = 0.02). The type of assessor was significant with general pediatricians having a longer median total wait time compared with developmental pediatricians, which was contrary to expectations. Further analysis showed that general pediatricians had a shorter median time 1 (4 versus 6 months for developmental pediatricians), and an identical median time 2 (1 month), though their combined time 1 and 2 was longer. The differences in total wait times between provinces were sufficiently significant to merit inclusion in the model, with total wait time varying from 5 months in Alberta to 14.5 months in Quebec. As type of assessor was significantly associated with time spent on assessment (Kruskal-Wallis chi-squared = 5.59; degrees of freedom [d.f.] = 2; *p* = 0.003), these two variables were not tested together in the multiple regression model. In the multi-variate model, time spent on assessment remained significantly associated with total wait time (β = 0.004, *p* = 0.002). It remained marginally significant when controlling for province (β = 0.003, *p* = 0.05), but province itself was not significantly associated with total wait time (partial *F* test = 0.88, *p* = 0.56). Type of assessor was not significantly associated with total wait time in the multi-variate model. The *R*^2^ for the full data set model was 0.17. The mean adjusted total wait time after back transformation was 8.5 months (95% confidence interval 7, 10). Adjusted values for the full data set are plotted in Fig. 3.

**Table 4:** Bivariate analyses of associations between continuous putative variables and total wait time

Continuous variables		Test (*r*_s_)	*p*
Time spent on assessment (minutes)		0.31	0.02^a^
Years in practice		−0.13	0.34
Categorical variables	Median wait time (months)	Test	p
Type of assessor		Kruskal-Wallis chi-squared = 3.66	0.16^a^
Developmental pediatrician	6.5		
General pediatrician	7.5		
Other	3		
Accepts referral from family doctor		Wilcoxon rank sum test *W* = 200	0.24
Yes	7		
No	6		
Practices in team		Wilcoxon rank sum test *W* = 308.5	0.25
Yes	9		
No	6.5		
Catchment		Kruskal-Wallis chi-squared = 2.85	0.42
Within city only	6.5		
Within regional health authority	9		
Within province/territory	6.5		
No defined catchment	6.5		
Province of practice		Kruskal-Wallis chi-squared = 13.67	0.19 ^a^
British Columbia	9.5		
Alberta	5		
Ontario	6.5		
Quebec	14.5		
New Brunswick	8.5		
Other^b^	7.5		

^a^Variable meets cutoff of *p* < 0.2 to be included in regression analysis

^b^“Other” is the median wait time for provinces with one respondent (Manitoba, Saskatchewan, Nova Scotia, Newfoundland and Labrador, Yukon, and Nunavut) that have been collapsed in the displayed results to prevent identification of individual respondents’ wait times

The total wait time was also analyzed based on use of the various diagnostic tools (Table 5). There were no significant differences in total wait time between respondents who did or did not use a particular tool. There was also no significant difference in total wait time between those who used diagnostic tools (median 7 months) and those who did not (median 6.3 months; Wilcoxon *W* = 295.5, *p* = 0.54).

**Table 5:** Wait times by diagnostic tool use

Test	*n* using tool	Median total wait among those endorsing use	Median total wait among those not endorsing use	*p*
ADOS	29	7.4	6.1	0.12
ADI-R	15	7	6.9	0.99
CARS	9	8	6.9	0.62
SRS	8	8.2	6.9	0.12
VABS	7	9.8	6.9	0.2
No test	12	6.3	7	0.54

Wait time reported in months; *p* value based on Wilcoxon estimate. *ADI-R* Autism Diagnostic Interview–Revised, *ADOS* Autism Diagnostic Observation Schedule, *CARS* Childhood Autism Rating Scale, *SRS* Social Responsiveness Scale, *VABS* Vineland Adaptive Behavior Scale

## Discussion

This study is the first to examine detailed self-reported practice patterns and wait times for ASD diagnosis among pediatricians across Canada. There was wide variation in reported practices for the diagnosis of ASD, including personnel and tools used in the assessment. There were no two identically composed MDTs across the country, which may reflect the lack of uniformity in guidance documents regarding the necessary personnel for ASD diagnostic assessment [4, 5, 9, 24].

A longer time spent on the assessment was significantly associated with longer total wait from referral to diagnosis, indicating that clinical decisions regarding necessary assessment elements have an important impact on wait times for families. Physicians with longer assessment times will likely have fewer available clinic slots and as a result will see fewer patients, lengthening their queue for ASD assessment. Use of a diagnostic tool was not significantly associated with total wait time, though statistical power may have limited our ability to detect a significant association, making this question worthy of further study. A wide range of reported wait times between the first clinic visit and the completion of the assessment was also observed. This period may represent a particularly stressful time for families as they likely know their child is being assessed for ASD but do not have the diagnosis required (in most jurisdictions) to access intervention. Each component of diagnostic delay may put the child at risk for suboptimal developmental outcomes [11].

Given the increase in prevalence of ASD diagnoses [2], demand for diagnostic assessments may exceed available resources, leading to wait times. As such, our results are relevant to all jurisdictions that provide publicly funded ASD diagnostic assessments. A study from the UK using data from 2001 to 2002 found that only 19% of assessments occurred within the recommended time frame of 30 weeks [25]. The time period between referral and receipt of ASD diagnosis has been repeatedly described as a highly stressful time for families, increasing the impetus to provide timely access to diagnosis [26, 27]. Parental stress and dissatisfaction are significantly associated with a higher number of professionals seen during the diagnostic process [26, 28]. To meet diagnostic demand, clinical guidelines for ASD diagnosis have focused on the need to train more providers to perform ASD assessments [4] and to fund more MDTs [5]. Our results suggest that further work is needed to determine the optimal balance between accuracy, quality, and efficiency in ASD assessments, allowing a higher volume of assessments to be completed and reducing wait times.

Our analysis revealed intriguing findings regarding the types of clinicians involved in the diagnostic assessment. Many pediatricians conducted developmental consultations (with wait times and billing costs) but did not provide ASD diagnoses in their practices. Though general pediatricians had a trend toward a shorter time to first visit of the diagnostic assessment, they trended toward a longer overall time to completion of the assessment. General pediatricians who diagnose ASD may avert the need for an additional referral to a developmental pediatrician or MDT in the diagnostic journey for families. Further work is needed to ensure that a general pediatrician diagnosis of ASD is accurate, acceptable to families, and that it is completed in an expedient manner. A qualitative study of general pediatricians in Ontario, Canada has shown varying willingness to diagnose ASD in their practices [29]. While many of those interviewed felt they were able to provide quality assessments that helped families access services faster than they would have if they waited for a subspecialist, there were identified barriers to conducting ASD diagnostic assessments, including uncertainty about the role of the general pediatrician in ASD diagnosis, inadequate training, and inadequate remuneration. In order to deal with diagnostic uncertainty, solo general pediatricians talked to other clinical staff in their office or reached out to subspecialist colleagues for “hallway consultations.” Similar to the results of this survey, participants reported differing use of diagnostic tools, including the ADOS, an abbreviated form of the ADOS, and using a screening tool such as the Modified Checklist for Autism in Toddlers [30] to structure their diagnostic interview. Participants identified barriers beyond identifying ASD, including disclosure of the diagnosis to families and knowledge of available resources in a fragmented system; as such, efforts to increase diagnostic capacity in general pediatricians must consider aspects of diagnostic assessment beyond accuracy, including communication skills and availability of service navigation.

A number of study limitations were present. The low response rate and the voluntary nature of the survey increase the potential for volunteer bias with respondents more likely to have an interest in ASD. As such, caution should be taken in extrapolating the results to all Canadian pediatricians. The study sample did not include psychologists and psychiatrists, who may also be involved in the diagnosis of ASD. Wait times in the survey were self-reported by clinicians, leading to the potential for reporting bias. To minimize the possibility of recall bias, participants were asked to report their current wait time, as opposed to estimating an average over a previous interval. Participants were carefully instructed in the consent form that they would be asked for their current wait time for ASD diagnostic assessment and to have this information prepared. This information could not be verified and is an acknowledged limitation of the work. The total wait time in this analysis only considered the wait time for one referral; this will underestimate wait times for diagnostic journeys that include non-productive referrals to non-diagnosing practitioners at early stages of the assessment pathway. The use of the single wait time for ASD diagnosis was chosen so that the influence of diagnostic practices on wait time would not be diluted by adding the wait times for other physician referrals. The total number of respondents limited the statistical power, though the response rate is similar to other Canadian physician surveys [31]. With a larger sample size, additional significant determinants of wait times may have been identified. The findings are nevertheless highly informative as they represent the first study looking at this critical question of capacity in the face of growing ASD prevalence.

Results of this study have identified an important association between the length of the ASD diagnostic assessment and wait times, although far more research is needed to determine the optimal balance between efficiency and comprehensiveness for a complex condition such as ASD. Further analysis is needed at the individual patient level, such as through health administrative or insurance databases, to determine the proportion of children/adults receiving their diagnoses from various providers/teams. This could be compared across jurisdictions with differing requirements for ASD diagnosis, including analysis of resource use and wait times. The variability in diagnostic assessment models reported in this study demands further evaluation of the accuracy of assessment types, such as MDT versus solo assessment and subspecialist versus general pediatrician assessment. Any system-wide strategy for improving efficiency of ASD diagnostic assessments should have accompanying qualitative examination related to uptake of new recommendations and requirements in all relevant stakeholders.

## Conclusion

In conclusion, Canadian pediatric practices for ASD diagnosis vary substantially. Assessment time is a significant determinant of total wait time for ASD diagnosis. Further work is needed to identify efficient assessment strategies that preserve reasonable accuracy and quality while allowing families to access timely diagnosis.

### Additional file

Additional file 1: Survey distributed to Canadian Paediatric Society. (DOCX 22 kb)

## Appendix 1

**Table 6:** Billing codes reported by participants who do not diagnose ASD

Billing codes used:	*n*	%
BC–00511 ($411.87)	2	5.9%
BC–00554 ($166.51)	1	2.9%
Alberta 03.08A CMXV30 ($229.15)	4	11.8%
Ontario–A265 ($167)	5	23.5%
Ontario–K122 ($80.30/30 min)	1	2.9%
Ontario–K123 ($91.10/30 min)	1	2.9%
Quebec–09165 ($187.25)	4	11.8%
Quebec–09127 ($56.65)	1	2.9%
Nova Scotia–03.08 ($171.82)	3	5.9%
Newfoundland–101 ($174.04	1	2.9%
Other^a^	2	5.9%
Do not know	2	5.9%

^a^Written responses: “Alternate payment” and “Psychosocial visit under remuneration mixte”

## Appendix 2

**Table 7:** Billing codes used in the ASD diagnostic assessment

Billing code	Amount	First visit (*n*)	Second visit (*n*)	Third visit (*n*)	Fourth visit (*n*)
Alberta-3.08A	$198.04	3	–	–	–
Alberta-03.08A CMXC30	$229.15	3	5	1	–
Alberta-03.08A + 03.08J	$198.04 + $59.41 per 15 min unit after 30 min	2	–	–	–
Alberta-03.08A CMXC30 + 03.08J	$229.15 + $59.41 per 15 min unit after 30 min	1	1	–	–
Alberta-03.03F	$99.02	–	2	–	–
Alberta-03.03F + 03.03FA	$99.02 + $59.41 per 15 min unit after 30 min	–	1	–	–
Alberta-03.03F CMXV30 + 03.03FA	$130.13 + $59.41 per 15 min unit after 30 min	–	1	–	–
BC-00511	$411.87	2	–	–	–
BC-00512	$99.19	–	–	1	–
BC-00554	$76.71	–	1	–	–
Manitoba-8552	$48.65 per 15 min unit (includes report writing)	1	–	–	–
New Brunswick-14.1-93	$217	3	–	–	–
New Brunswick-14.1-94	$133	–	1	–	–
New Brunswick-14.2-85	$92.40	–	–	1	–
New Brunswick-14.8C-91	$263.20	–	1	–	–
Newfoundland-101	$174.04	1	–	–	–
Newfoundland-113	$93.37	–	1	–	–
Nova Scotia-03.08	$171.82	1	–	–	–
Ontario-A265	$167	10	–	–	–
Ontario-A667	$395.65	10	–	–	–
Ontario-A260	$300.70	3	1	1	–
Ontario-A2662	$395.65	2	–	–	–
Ontario-A2661	$68.80	0	1	–	–
Ontario-K119	$100.00	1	–	–	–
Ontario-K122	$80.30 per 30 min unit	–	4	1	–
Ontario-K123	$91.10 per 30 min unit	–	12	5	2
Quebec-09127	$57	–	–	–	–
Quebec-09165	$187	2	1	1	–
Quebec-09129	$47	1	–	–	–
Quebec-15164	$55.05 per 15 min unit	–	2	1	–
Saskatchewan-9C	$125	1	–	–	–
Saskatchewan-3C	$89.40	–	1	–	–
Nunavut K1/K2	(unable to determine)	1	1	1	–
NB-90 + 2172	(unable to determine)	–	1	–	–
Do not know		5	8	1	–
Alternate funding plan		3	2	1	1
No response		1	1	1	1
Total		57	49	16	3

The number of participants indicating use of each billing code or billing code combination is displayed. The fifth clinic visit and any additional visits are not displayed and are described in the text. Where the respondent selected more than one billing code per visit but only one of those selected could be used for a patient visit according to the province’s fee schedule, the billing code with the higher amount was included in the analysis

## Appendix 3

**Table 8:** Time spent administering and scoring diagnostic tools

Tools	Number using tool	Median (min)	Range (min)	Interquartile range (min)
Autism Diagnostic Observation Schedule (ADOS)	29	60	30–120	60–79
Autism Diagnostic Interview–Revised (ADI-R)	15	90	20–120	53–105
Childhood Autism Rating Scale	9	20	15–60	15–30
Social Responsiveness Scale	8	10	5–45	10–15
Vineland Adaptive Behavior Scales	7	45	18–90	38–60
Social Communication Questionnaire	4	23	10–45	18–30
M-CHAT	4	NS	–	–
Adaptive Behavior Assessment System	2	10	–	–
“DSM-5 criteria”	2	NS	–	–
“Cognitive assessment”	2	90	–	–
Mullen Scales of Early Learning	1	60	–	–
“Neurodevelopmental assessment”	1	45	–	–
“Academic screen”	1	40	–	–
Peabody Picture Vocabulary Test	1	30	–	–
Beery-Butenica Test of Visual-Motor Integration	1	20	–	–
Bayley Scales of Infant Development	1	NS	–	–
Diagnostic Interview for Social and Communication Disorders	–	–	–	–
None	12			

*NS* not specified, responses appearing in quotations are written-in responses not corresponding with an identified tool

## Abbreviations

ADI-R: Autism Diagnostic Interview–Revised
ADOS: Autism Diagnostic Observation Schedule
ASD: Autism spectrum disorder
CARS: Childhood Autism Rating Scale
CPS: Canadian Pediatric Society
d.f.: Degrees of freedom
ln: Natural logarithm
MDT: Multidisciplinary team
PEI: Prince Edward Island
REDCap: Research Electronic Data Capture
USA: United States of America
