# Supplementary material for: Practice patterns and determinants of wait time for autism spectrum disorder diagnosis in Canada
Source: Mol Autism. 2018 Mar 6;9:16. doi: 10.1186/s13229-018-0201-0 (PMC5840789; doi:10.1186/s13229-018-0201-0)
Supplement: Supplementary file 1 — Survey distributed to Canadian Paediatric Society. (DOCX 22 kb) [file 13229_2018_201_MOESM1_ESM.docx]

**Additional file 1: Survey distributed to Canadian Paediatric Society**

1. What is your age? _______
2. What is your sex? (M/F)
3. In which province/territory do you practice?
   1. Alberta
   2. British Columbia
   3. Manitoba
   4. New Brunswick
   5. Newfoundland & Labrador
   6. Nova Scotia
   7. Northwest Territories
   8. Nunavut
   9. Ontario
   10. Prince Edward Island
   11. Quebec
   12. Saskatchewan
   13. Yukon Territory
4. How large is your catchment area?
   1. Within-city only
   2. Within regional health authority
   3. Within province/territory
   4. No defined catchment
5. How many years have you been in paediatric practice? _________
6. What type of health professional are you?
   1. General paediatrician
   2. Developmental paediatrician
   3. Psychiatrist
   4. Psychologist
   5. Other_______________
7. Do you have any formal training in child development or autism spectrum disorder (examples: subspecialty residency or fellowship in developmental paediatrics)? (Y/N)
   1. If Yes, please describe: _________________________________________
8. Do you give a diagnosis of autism spectrum disorder in your practice? (Y/N)

**IF YES, go to question 9**

**IF NO, go to question 23**

- 1. **(Only if participant selects general paediatrician for question 6 AND Yes to question 8)** Of the cases with suspected autism spectrum disorder in your practice, for what percentage do you provide a definitive diagnostic assessment (versus referring for subspecialist assessment)?Select one:
     1. 0-25%
     2. 26-50%
     3. 51-75%
     4. 76-100%

1. Do you accept referrals from family doctors for autism spectrum disorder diagnostic assessment (Y/N)?
2. How many clinic visits does it take you (on average) to perform an autism spectrum disorder diagnostic assessment and communicate the results?
   1. 1
   2. 2
   3. 3
   4. 4
   5. 5
   6. More than 5
3. Which billing code(s) do you use for the first visit?
   1. Alberta
   2. BC – 00511
   3. BC – 00512
   4. BC – 00514
   5. BC – 00554
   6. Manitoba – 8540
   7. Manitoba – 8552
   8. Manitoba – 8404
   9. Manitoba – 8555
   10. New Brunswick – 14.1-93
   11. New Brunswick – 14.1-94
   12. New Brunswick – 14.2-85
   13. New Brunswick – 14.2-86
   14. New Brunswick – 14.8C-91
   15. New Brunswick – 14.8-239
   16. New Brunswick – 14.8-194
   17. Newfoundland – 101
   18. Newfoundland – 102
   19. Newfoundland - 112
   20. Newfoundland – 113
   21. Newfoundland – 114
   22. Newfoundland – 115
   23. Newfoundland – 144
   24. Newfoundland – 181
   25. NWT – PA001
   26. NWT – PA007
   27. NWT – PA016
   28. Nova Scotia – 03.08
   29. Nunavut – A-1
   30. Nunavut – A-3
   31. Ontario – A265
   32. Ontario – A260
   33. Ontario – A667
   34. Ontario – K119
   35. Ontario – K122
   36. Ontario – K123
   37. PEI – 1160
   38. PEI – 1162
   39. PEI – 1110
   40. PEI – 1111
   41. PEI – 1112
   42. PEI – 2507
   43. PEI – 2586
   44. Quebec – 09127
   45. Quebec – 09165
   46. Quebec – 09129
   47. Quebec – 15164
   48. Saskatchewan – 14C
   49. Saskatchewan – 9C
   50. Saskatchewan – 11C
   51. Saskatchewan – 3C
   52. Yukon – 0510
   53. Yukon – 0511
   54. Yukon – 0512
   55. Yukon – 0514
   56. Yukon – 0550
   57. Yukon – 0551
   58. Yukon – 0554
   59. Other (please list and indicate your province): ___________
   60. Other (please list and indicate your province): ___________
   61. Other (please list and indicate your province): ____________
   62. Don’t know
4. (If the participant indicates two or more clinic visits) Which billing code(s) do you use for the second visit?
   1. Alberta – 3.0 PED CMXV30
   2. Alberta – 3.0 PED CMXV35
   3. BC – 00511
   4. BC – 00512
   5. BC – 00514
   6. BC – 00554
   7. Manitoba – 8540
   8. Manitoba – 8552
   9. Manitoba – 8404
   10. Manitoba – 8555
   11. New Brunswick – 14.1-93
   12. New Brunswick – 14.1-94
   13. New Brunswick – 14.2-85
   14. New Brunswick – 14.2-86
   15. New Brunswick – 14.8C-91
   16. New Brunswick – 14.8-239
   17. New Brunswick – 14.8-194
   18. Newfoundland – 101
   19. Newfoundland – 102
   20. Newfoundland - 112
   21. Newfoundland – 113
   22. Newfoundland – 114
   23. Newfoundland – 115
   24. Newfoundland – 144
   25. Newfoundland – 181
   26. NWT – PA001
   27. NWT – PA007
   28. NWT – PA016
   29. Nova Scotia – 03.08
   30. Nunavut – A-1
   31. Nunavut – A-3
   32. Ontario – A265
   33. Ontario – A260
   34. Ontario – A667
   35. Ontario – K119
   36. Ontario – K122
   37. Ontario – K123
   38. PEI – 1160
   39. PEI – 1162
   40. PEI – 1110
   41. PEI – 1111
   42. PEI – 1112
   43. PEI – 2507
   44. PEI – 2586
   45. Quebec – 09127
   46. Quebec – 09165
   47. Quebec – 09129
   48. Quebec – 15164
   49. Saskatchewan – 14C
   50. Saskatchewan – 9C
   51. Saskatchewan – 11C
   52. Saskatchewan – 3C
   53. Yukon – 0510
   54. Yukon – 0511
   55. Yukon – 0512
   56. Yukon – 0514
   57. Yukon – 0550
   58. Yukon – 0551
   59. Yukon – 0554
   60. Other (please list and indicate your province): ___________
   61. Other (please list and indicate your province): ___________
   62. Other (please list and indicate your province): ___________
   63. Don’t know
5. (If the participant indicates three or more clinic visits) Which billing code(s) do you use for the third visit?
   1. Alberta – 3.0 PED CMXV30
   2. Alberta – 3.0 PED CMXV35
   3. BC – 00511
   4. BC – 00512
   5. BC – 00514
   6. BC – 00554
   7. Manitoba – 8540
   8. Manitoba – 8552
   9. Manitoba – 8404
   10. Manitoba – 8555
   11. New Brunswick – 14.1-93
   12. New Brunswick – 14.1-94
   13. New Brunswick – 14.2-85
   14. New Brunswick – 14.2-86
   15. New Brunswick – 14.8C-91
   16. New Brunswick – 14.8-239
   17. New Brunswick – 14.8-194
   18. Newfoundland – 101
   19. Newfoundland – 102
   20. Newfoundland - 112
   21. Newfoundland – 113
   22. Newfoundland – 114
   23. Newfoundland – 115
   24. Newfoundland – 144
   25. Newfoundland – 181
   26. NWT – PA001
   27. NWT – PA007
   28. NWT – PA016
   29. Nova Scotia – 03.08
   30. Nunavut – A-1
   31. Nunavut – A-3
   32. Ontario – A265
   33. Ontario – A260
   34. Ontario – A667
   35. Ontario – K119
   36. Ontario – K122
   37. Ontario – K123
   38. PEI – 1160
   39. PEI – 1162
   40. PEI – 1110
   41. PEI – 1111
   42. PEI – 1112
   43. PEI – 2507
   44. PEI – 2586
   45. Quebec – 09127
   46. Quebec – 09165
   47. Quebec – 09129
   48. Quebec – 15164
   49. Saskatchewan – 14C
   50. Saskatchewan – 9C
   51. Saskatchewan – 11C
   52. Saskatchewan – 3C
   53. Yukon – 0510
   54. Yukon – 0511
   55. Yukon – 0512
   56. Yukon – 0514
   57. Yukon – 0550
   58. Yukon – 0551
   59. Yukon – 0554
   60. Other (please list and indicate your province): ___________
   61. Other (please list and indicate your province): ___________
   62. Other (please list and indicate your province): ___________
   63. Don’t know
6. (If the participant indicates four or more clinic visits) Which billing code(s) do you use for the fourth visit?
   1. Alberta – 3.0 PED CMXV30
   2. Alberta – 3.0 PED CMXV35
   3. BC – 00511
   4. BC – 00512
   5. BC – 00514
   6. BC – 00554
   7. Manitoba – 8540
   8. Manitoba – 8552
   9. Manitoba – 8404
   10. Manitoba – 8555
   11. New Brunswick – 14.1-93
   12. New Brunswick – 14.1-94
   13. New Brunswick – 14.2-85
   14. New Brunswick – 14.2-86
   15. New Brunswick – 14.8C-91
   16. New Brunswick – 14.8-239
   17. New Brunswick – 14.8-194
   18. Newfoundland – 101
   19. Newfoundland – 102
   20. Newfoundland - 112
   21. Newfoundland – 113
   22. Newfoundland – 114
   23. Newfoundland – 115
   24. Newfoundland – 144
   25. Newfoundland – 181
   26. NWT – PA001
   27. NWT – PA007
   28. NWT – PA016
   29. Nova Scotia – 03.08
   30. Nunavut – A-1
   31. Nunavut – A-3
   32. Ontario – A265
   33. Ontario – A260
   34. Ontario – A667
   35. Ontario – K119
   36. Ontario – K122
   37. Ontario – K123
   38. PEI – 1160
   39. PEI – 1162
   40. PEI – 1110
   41. PEI – 1111
   42. PEI – 1112
   43. PEI – 2507
   44. PEI – 2586
   45. Quebec – 09127
   46. Quebec – 09165
   47. Quebec – 09129
   48. Quebec – 15164
   49. Saskatchewan – 14C
   50. Saskatchewan – 9C
   51. Saskatchewan – 11C
   52. Saskatchewan – 3C
   53. Yukon – 0510
   54. Yukon – 0511
   55. Yukon – 0512
   56. Yukon – 0514
   57. Yukon – 0550
   58. Yukon – 0551
   59. Yukon – 0554
   60. Other (please list and indicate your province): ___________
   61. Other (please list and indicate your province): ___________
   62. Other (please list and indicate your province): ___________
   63. Don’t know
7. (If the participant indicates five or more clinic visits) Which billing code(s) do you use for the fifth visit?
   1. Alberta – 3.0 PED CMXV30
   2. Alberta – 3.0 PED CMXV35
   3. BC – 00511
   4. BC – 00512
   5. BC – 00514
   6. BC – 00554
   7. Manitoba – 8540
   8. Manitoba – 8552
   9. Manitoba – 8404
   10. Manitoba – 8555
   11. New Brunswick – 14.1-93
   12. New Brunswick – 14.1-94
   13. New Brunswick – 14.2-85
   14. New Brunswick – 14.2-86
   15. New Brunswick – 14.8C-91
   16. New Brunswick – 14.8-239
   17. New Brunswick – 14.8-194
   18. Newfoundland – 101
   19. Newfoundland – 102
   20. Newfoundland - 112
   21. Newfoundland – 113
   22. Newfoundland – 114
   23. Newfoundland – 115
   24. Newfoundland – 144
   25. Newfoundland – 181
   26. NWT – PA001
   27. NWT – PA007
   28. NWT – PA016
   29. Nova Scotia – 03.08
   30. Nunavut – A-1
   31. Nunavut – A-3
   32. Ontario – A265
   33. Ontario – A260
   34. Ontario – A667
   35. Ontario – K119
   36. Ontario – K122
   37. Ontario – K123
   38. PEI – 1160
   39. PEI – 1162
   40. PEI – 1110
   41. PEI – 1111
   42. PEI – 1112
   43. PEI – 2507
   44. PEI – 2586
   45. Quebec – 09127
   46. Quebec – 09165
   47. Quebec – 09129
   48. Quebec – 15164
   49. Saskatchewan – 14C
   50. Saskatchewan – 9C
   51. Saskatchewan – 11C
   52. Saskatchewan – 3C
   53. Yukon – 0510
   54. Yukon – 0511
   55. Yukon – 0512
   56. Yukon – 0514
   57. Yukon – 0550
   58. Yukon – 0551
   59. Yukon – 0554
   60. Other (please list and indicate your province): ___________
   61. Other (please list and indicate your province): ___________
   62. Other (please list and indicate your province): ___________
   63. Don’t know
8. (If the participant indicates more than five clinic visits) Which billing code(s) do you use in most cases for subsequent ASD diagnostic visits?
   1. Alberta – 3.0 PED CMXV30
   2. Alberta – 3.0 PED CMXV35
   3. BC – 00511
   4. BC – 00512
   5. BC – 00514
   6. BC – 00554
   7. Manitoba – 8540
   8. Manitoba – 8552
   9. Manitoba – 8404
   10. Manitoba – 8555
   11. New Brunswick – 14.1-93
   12. New Brunswick – 14.1-94
   13. New Brunswick – 14.2-85
   14. New Brunswick – 14.2-86
   15. New Brunswick – 14.8C-91
   16. New Brunswick – 14.8-239
   17. New Brunswick – 14.8-194
   18. Newfoundland – 101
   19. Newfoundland – 102
   20. Newfoundland - 112
   21. Newfoundland – 113
   22. Newfoundland – 114
   23. Newfoundland – 115
   24. Newfoundland – 144
   25. Newfoundland – 181
   26. NWT – PA001
   27. NWT – PA007
   28. NWT – PA016
   29. Nova Scotia – 03.08
   30. Nunavut – A-1
   31. Nunavut – A-3
   32. Ontario – A265
   33. Ontario – A260
   34. Ontario – A667
   35. Ontario – K119
   36. Ontario – K122
   37. Ontario – K123
   38. PEI – 1160
   39. PEI – 1162
   40. PEI – 1110
   41. PEI – 1111
   42. PEI – 1112
   43. PEI – 2507
   44. PEI – 2586
   45. Quebec – 09127
   46. Quebec – 09165
   47. Quebec – 09129
   48. Quebec – 15164
   49. Saskatchewan – 14C
   50. Saskatchewan – 9C
   51. Saskatchewan – 11C
   52. Saskatchewan – 3C
   53. Yukon – 0510
   54. Yukon – 0511
   55. Yukon – 0512
   56. Yukon – 0514
   57. Yukon – 0550
   58. Yukon – 0551
   59. Yukon – 0554
   60. Other (please list and indicate your province): ___________
   61. Other (please list and indicate your province): ___________
   62. Other (please list and indicate your province): ___________
   63. Don’t know
9. How long is each clinic visit (on average)?
   1. 30 minutes or less
   2. 31-60 minutes
   3. 61-90 minutes
   4. 91-120 minutes
   5. 121-180 minutes
   6. > 180 minutes
10. Which tools do you use during the diagnostic assessment for autism spectrum disorder IN MOST CASES? Please check off the tool and indicate how many minutes it takes you to complete it (including scoring):
    1. Autism Diagnostic Interview – Revised: completion time (minutes)____________
    2. Autism Diagnostic Observation Schedule – 2 _____________
    3. Social Responsiveness Scale: _______________
    4. Social Communication Questionnaire: ______________
    5. Vineland Adaptive Behaviour Scales 2^nd^ ed.: _____________
    6. Mullen Scales of Early Learning: _____________
    7. Diagnostic Interview for Social and Communication Disorders: ______________
    8. Childhood Autism Rating Scale: _______________
    9. Other (specify): _________________________ (time) ____________________
    10. None
11. Do you conduct autism spectrum disorder diagnostic assessments as part of a multi-disciplinary team (defined as an assessment in collaboration with at least one other professional in a profession other than your own)? (Y/N) **(if No, please skip to question 17)**
12. For what per cent of cases do you provide diagnoses as part of a multidisciplinary team?
    1. 1-25%
    2. 26-50%
    3. 51-75%
    4. 76-100%
13. Which allied health professionals are available to your team? Include your role. Select all that apply.
    1. Psychology
    2. Speech-language pathology
    3. Occupational therapy
    4. Physiotherapy
    5. Social work
    6. Psychometry
    7. Clinical Genetics
    8. General Paediatrics
    9. Developmental Paediatrics
    10. Neurology
    11. Gastroenterology
    12. Behavioural Therapy
    13. Other: ____________________
14. Which professionals participate in the majority of the cases? Include your role. Select all that apply.
    1. Psychology
    2. Speech-language pathology
    3. Occupational therapy
    4. Physiotherapy
    5. Social work
    6. Psychometrist
    7. Clinical Genetics
    8. General Paediatrics
    9. Developmental Paediatrics
    10. Neurology
    11. Gastroenterology
    12. Behavioural Therapy
    13. Other: ______________________
15. For what per cent of cases do you consult with speech-language pathologists from outside of your institution (or use their assessment report) to help with your diagnosis (example: regional preschool Speech and Language Program)?
    1. 0-25%
    2. 26-50%
    3. 51-75%
    4. 76-100%
16. For what per cent of cases do you consult with occupational therapists from outside of your institution (or use their assessment report) to help with your diagnosis?
    1. 0-25%
    2. 26-50%
    3. 51-75%
    4. 76-100%
17. For what per cent of cases do you consult with early interventionists from outside of your institution (or use their assessment report) to help with your diagnosis?
    1. 0-25%
    2. 26-50%
    3. 51-75%
    4. 76-100%
18. How many months is the current wait time (from the time your practice receives the referral) to be seen for the first visit of the diagnostic assessment? ____________
19. How many weeks is the time period from your first clinic assessment visit to the communication of a diagnosis of autism spectrum disorder by you to the family? _________
20. Which of the following tests would you routinely offer when making a diagnosis of ASD? Select all that apply.
    1. Chromosomal microarray
    2. Fragile X testing
    3. Hearing assessment
    4. Vision assessment
    5. MECP2 for Rett Syndrome
    6. Metabolic screening
    7. MRI brain
    8. EEG
    9. Other________________________
    10. None

**STOP POINT FOR PARTICIPANTS WHO DIAGNOSE ASD**

1. How many weeks is the current wait time from receipt of a referral for a child with developmental concerns to you seeing the child in clinic? ___________
2. How many minutes do you schedule for a first clinic visit for a child with developmental concerns?
   1. 15 minutes or less
   2. 16-30 minutes
   3. 31-45 minutes
   4. 46-60 minutes
   5. 61-75 minutes
   6. 76-90 minutes
   7. More than 90 minutes
3. Which billing code(s) do you use when seeing a child referred with developmental concerns?
   1. Alberta – 3.0 PED CMXV30
   2. Alberta – 3.0 PED CMXV35
   3. BC – 00511
   4. BC – 00512
   5. BC – 00514
   6. BC – 00554
   7. Manitoba – 8540
   8. Manitoba – 8552
   9. Manitoba – 8404
   10. Manitoba – 8555
   11. New Brunswick – 14.1-93
   12. New Brunswick – 14.1-94
   13. New Brunswick – 14.2-85
   14. New Brunswick – 14.2-86
   15. New Brunswick – 14.8C-91
   16. New Brunswick – 14.8-239
   17. New Brunswick – 14.8-194
   18. Newfoundland – 101
   19. Newfoundland – 102
   20. Newfoundland - 112
   21. Newfoundland – 113
   22. Newfoundland – 114
   23. Newfoundland – 115
   24. Newfoundland – 144
   25. Newfoundland – 181
   26. NWT – PA001
   27. NWT – PA007
   28. NWT – PA016
   29. Nova Scotia – 03.08
   30. Nunavut – A-1
   31. Nunavut – A-3
   32. Ontario – A265
   33. Ontario – A260
   34. Ontario – A667
   35. Ontario – K119
   36. Ontario – K122
   37. Ontario – K123
   38. PEI – 1160
   39. PEI – 1162
   40. PEI – 1110
   41. PEI – 1111
   42. PEI – 1112
   43. PEI – 2507
   44. PEI – 2586
   45. Quebec – 09127
   46. Quebec – 09165
   47. Quebec – 09129
   48. Quebec – 15164
   49. Saskatchewan – 14C
   50. Saskatchewan – 9C
   51. Saskatchewan – 11C
   52. Saskatchewan – 3C
   53. Yukon – 0510
   54. Yukon – 0511
   55. Yukon – 0512
   56. Yukon – 0514
   57. Yukon – 0550
   58. Yukon – 0551
   59. Yukon – 0554
   60. Other (please list and indicate your province): ___________
   61. Other (please list and indicate your province): ___________
   62. Other (please list and indicate your province): ___________
   63. Don't know

**STOP POINT FOR PARTICIPANTS WHO DO NOT DIAGNOSE ASD**
